# Supplementary material for: Attention expedites target selection by prioritizing the neural processing of distractor features
Source: Commun Biol. 2021 Jun 29;4:814. doi: 10.1038/s42003-021-02305-9 (PMC8242025; doi:10.1038/s42003-021-02305-9)
Supplement: Supplementary file 2 — Supplementary Information [file 42003_2021_2305_MOESM2_ESM.pdf]

Supplementary Information

Attention expedites target selection by prioritizing the neural processing of distractor features

Mandy V. Bartsch, Christian Merkel, Mircea A. Schoenfeld, Jens-Max Hopf

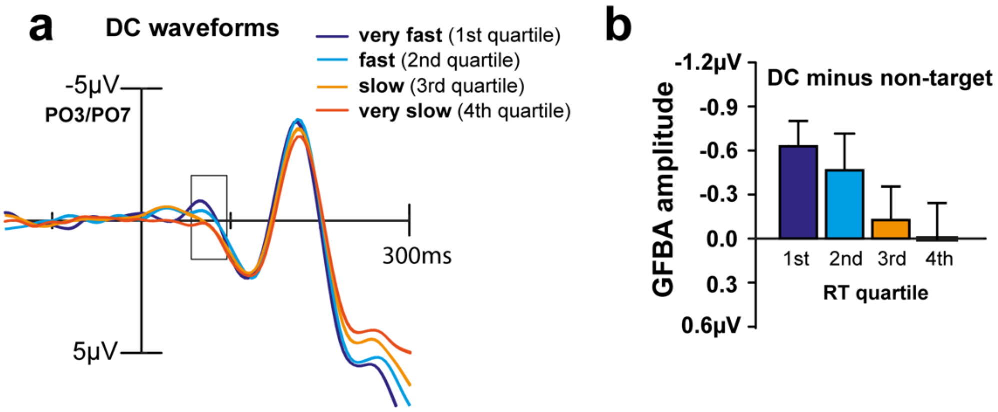

Supplementary Figure 1. Quartile response time (RT) split for DC trials. *The quartile RT split was performed analogous to the median RT split as detailed in the Methods section of the manuscript.* ***(a)*** *ERP waveforms for DC trials (probe matches distracting target color alternative) split into four quartiles (very fast, fast, slow, and very slow RTs). As can be seen, in the time range of the early distractor negativity (black rectangle), the response shows an increasing negativity with decreasing RT.* ***(b)*** *Mean GFBA amplitudes (DC minus non-target differences) of the quartile responses in the time range marked by the black rectangle in (a) (57-96ms). The early modulation effect is gradually reduced in amplitude with increasing response time, with a large amplitude effect appearing in very fast (1st quartile, dark blue) and a smaller effect in fast trials (2nd quartile, light blue). The early DC modulation is minimal in slow (3rd quartile, orange) and absent in very slow trials (4th quartile), indicating that it is primarily driven by fast target discriminations. The error bars represent the standard error of the mean (SEM).*

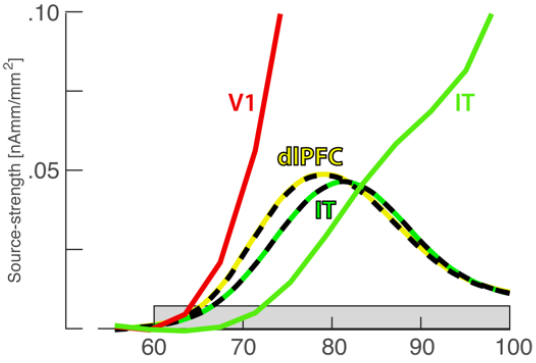

Supplementary Figure 2. Direct comparison of stimulus-elicited feedforward activity (Figure 4a) with the early distractor selection (Figure 4b) in the 60-100ms time range. *The stimulus-elicited source waves in V1 and IT (red-solid, green-solid) are replotted together with dlPFC and IT source waves (yellow-dashed, green-dashed) underlying the very early distractor selection (DC minus non-target) at the scale of Figure 4b. To compensate for vertical noise offsets, all responses are set to 0 between 55-60 ms. As can be seen, the mere stimulus-elicited feedforward response (solid waveforms) is far stronger than that underlying the early distractor selection (DC minus non-target difference waveforms, dashed). When depicted at the same scale, it becomes obvious that the dlPFC activity arises after the onset of the V1 feedforward response but with a very short delay.*

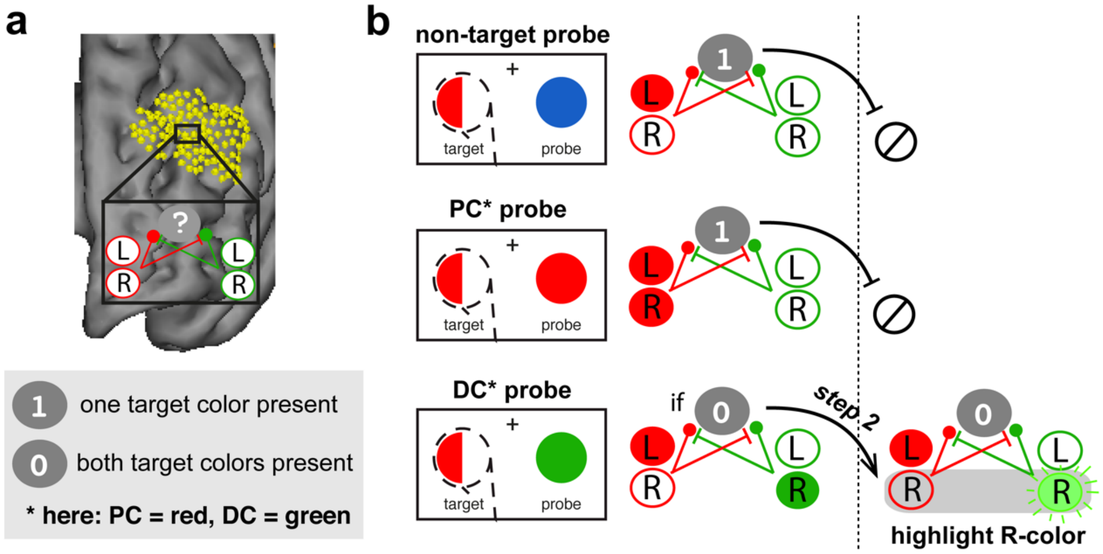

Supplementary Figure 3. Schematic illustration of a potential mechanism to facilitate rapid DC selection at the probe location. (a) *Let the dlPFC contain units with learned color-selectivity (here: red and green), with some preferring left visual field (VF) targets (L) and some preferring right VF targets (R). Among those implement a competitive link (opponent coding) between red and green units (|red-minus-green|).* ***(b)*** *Different states of competition signalling the presence of one or two target colors exemplified for trials with a red target in the left VF (Left-red units always active, filled red) and a probe in the right VF. Exemplary stimulus displays underlying the different competition states are shown on the left. For a* ***non-target probe****, only the target-red unit is active with the competition reporting 1. For a* ***PC probe,*** *the left- and right-preferring red units are active, but no green unit is active, the competition reports 1. For a* ***DC probe,*** *left-preferring red and right-preferring green units are active, with the competition reporting 0. When the competition is 0, highlight the color reported by the active units preferring the right VF (step 2). Importantly, as the competitive link is highly trained, the decision process can operate entirely on the feedforward response of color units to accomplish a rapid DC selection at the location of the probe. Note, the decision process is time-efficient as it does not require to access the identity of the colors presented at the target and probe side.*

Supplementary Note 1: Brain responses to non-target magenta-colored probes

In a given experimental block there were always three different non-target colors which appeared with the same frequency on the probe side. Two of them (either red and green, or blue and yellow) were used as target colors on different trial blocks. A third color (magenta) served as a non-target color but never defined the target. Those magenta probes seem to be a more "neutral" baseline for the target colors than the non-target colors that served as target on different trial blocks. However, because magenta never serves as a target color, the derivation of the probe-response (DC minus non-target, PC minus non-target) would always involve a comparison between different colors, i.e., confound the attention-related response with response differences due to color per se. Put differently, with magenta never being the target, there can be no "attend" minus "not attend" difference for magenta probes, that would eliminate low level effects of this color. Moreover, magenta was the non-target color that never defined the target in each subject. As a consequence, the response to magenta could be very specific for that color. Nevertheless, **Supplementary Figure 4** plots the brain response to magenta probes together with the response to the other non-target colors. As

can be seen, in the time-range of our main analysis (black rectangles) the brain response did not significantly differ between magenta and the other non-target colors. However, irrespective of the task, non-target colors that were used as the target color on other blocks show some negative enhancement between 100-200ms relative to magenta that never served as target color (see Supplementary Figure 4b). As outline above, it is difficult to know whether this enhancement reflects some sort of general task-relevance gained by the non-target colors that previously served as target, or a mere magenta-specific color-response.

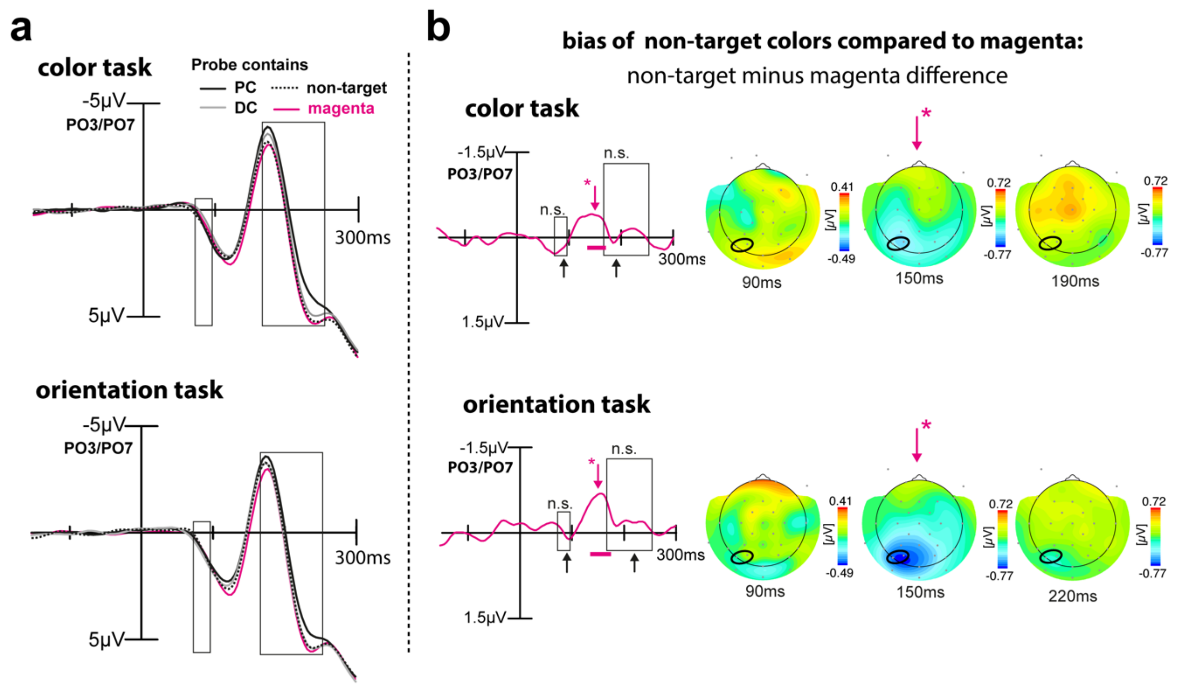

Supplementary Figure 4. ERP results for non-target magenta-colored probes. (a) *Shown is the ERP elicited by the probe at PO3/PO7 (signal averaged) for PC, DC, and non-target probes (replotted from Figure 3 and 7) together with the ERP elicited by non-target magenta probes when participants were to discriminate the color (upper row) or orientation (lower row) of the target. Rectangles highlight time ranges of significant brain response variations as defined by a 2x3 rANOVA (see Methods, main text). As can be seen, the brain response to magenta probes (magenta line) is quite similar to that of the other non-target colors (black dashed).* ***(b)*** *Difference waveforms of non-target minus magenta for the color (upper row) and orientation task (lower row) reveal a greater brain response (negativity) for non-target colors peaking around 150ms. Irrespective of the task, sliding-window t-tests (sample-by-sample, 11.8ms window between 0-300ms) confirm a significant difference of non-target and magenta between about 136-170ms after stimulus onset (magenta horizontal bars) but find no significant differences in the time ranges of our main analysis (rectangles). The respective topographical maps on the right display the field distribution at representative time points (early, middle, late). The positions of electrodes used for the analyses are highlighted (black ellipses). The magenta-colored non-target probes do not qualitatively differ from the other non-target colors in the early and late modulation time ranges of our main analysis. However, around 150ms, the non-target colors show some negative enhancement relative to magenta, potentially due to being task-relevant on other experimental blocks.*

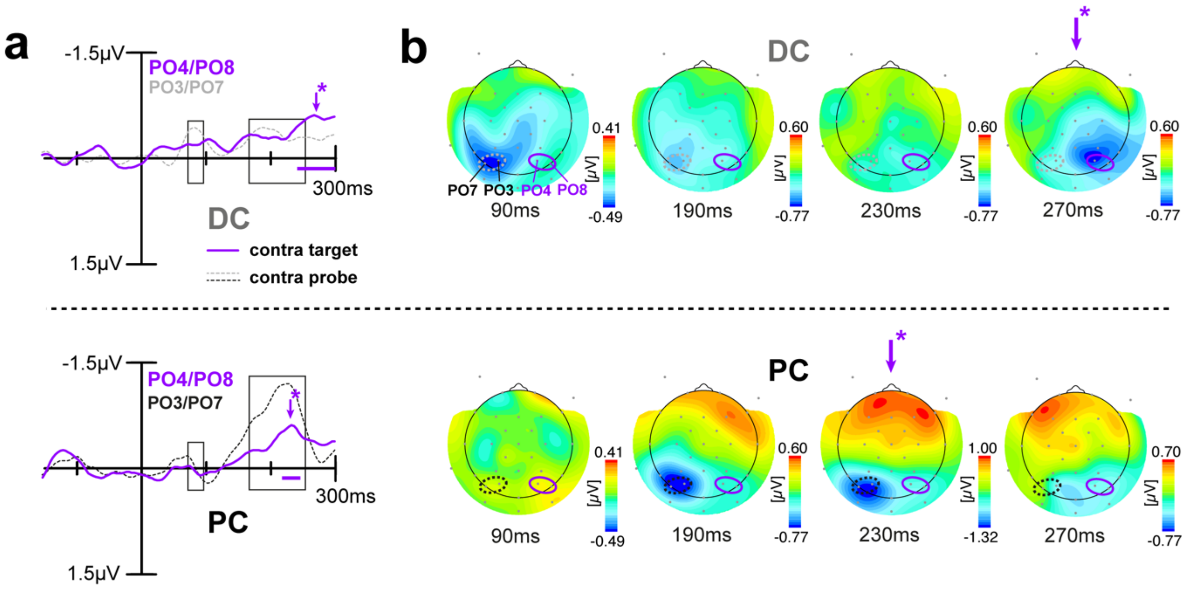

Supplementary Figure 5. ERP results for the color task: DC/PC minus non-target differences contra target. (a) *Shown are the ERP waveforms elicited by the target at PO4/PO8 (signal averaged, purple line) for the DC minus non-target (upper row) and PC minus non-target difference (lower row). Rectangles highlight time ranges of significant brain response variations contra probe as derived by the 2x3 rANOVA in the main analysis. For a better comparison, the ERP waveforms contra target are shown together with the respective ERPs contra probe (PO3/PO7, signal averaged, thin dashed grey or black line) replotted from Figure 3b, c. Contra target, a sliding window t-test (sample-by-sample, 11.8ms window) between 0-300ms revealed only significant modulations (p<0.02 for more than five consecutive time samples, see Methods) in late time ranges (purple horizontal bars, DC: 242-300ms, PC: 218-246ms).* ***(b)*** *The respective topographical field maps on the right display representative time points at either DC or PC modulation maxima, positions of electrodes used for the analyses are highlighted (dashed grey or black and purple ellipses). On DC trials (upper row), there is a prominent response maximum contra target at 270ms, which might reflect a delayed target processing. For PC trials (lower row), the significant modulation contra target seen in the ERP waveform around 230ms represents a spill of activity from the strong contra probe modulation (cf. field distribution at 230ms). Notably, there is no significant negativity contra target in the early time range neither on DC nor PC trials. Hence, the early biasing exclusively appears on DC trials contra probe, i.e., for the distracting alternative target color.*
