# Supplementary material for: Attention expedites target selection by prioritizing the neural processing of distractor features
Source: Commun Biol. 2021 Jun 29;4:814. doi: 10.1038/s42003-021-02305-9 (PMC8242025; doi:10.1038/s42003-021-02305-9)
Supplement: Supplementary file 3 — Reporting Summary [file 42003_2021_2305_MOESM3_ESM.pdf]

nature research

Corresponding author(s): Mandy V. Bartsch

Last updated by author(s): May 27, 2021

Reporting Summary

Nature Research wishes to improve the reproducibility of the work that we publish. This form provides structure for consistency and transparency in reporting. For further information on Nature Research policies, see our Editorial Policies and the Editorial Policy Checklist.

Statistics

For all statistical analyses, confirm that the following items are present in the figure legend, table legend, main text, or Methods section.

n/a	Confirmed	
☐	☒	The exact sample size (n) for each experimental group/condition, given as a discrete number and unit of measurement
☐	☒	A statement on whether measurements were taken from distinct samples or whether the same sample was measured repeatedly
☐	☒	The statistical test(s) used AND whether they are one- or two-sided<br>*Only common tests should be described solely by name; describe more complex techniques in the Methods section.*
☒	☐	A description of all covariates tested
☐	☒	A description of any assumptions or corrections, such as tests of normality and adjustment for multiple comparisons
☐	☒	A full description of the statistical parameters including central tendency (e.g. means) or other basic estimates (e.g. regression coefficient) AND variation (e.g. standard deviation) or associated estimates of uncertainty (e.g. confidence intervals)
☐	☒	For null hypothesis testing, the test statistic (e.g. F, t, r) with confidence intervals, effect sizes, degrees of freedom and P value noted<br>*Give P values as exact values whenever suitable.*
☒	☐	For Bayesian analysis, information on the choice of priors and Markov chain Monte Carlo settings
☒	☐	For hierarchical and complex designs, identification of the appropriate level for tests and full reporting of outcomes
☒	☐	Estimates of effect sizes (e.g. Cohen's d, Pearson's r), indicating how they were calculated

Our web collection on statistics for biologists contains articles on many of the points above.

Software and code

Policy information about availability of computer code

Data collection	MSI Software of the BTI Magnes 3600 recording system, Presentation® software (Neurobehavioral Systems Inc., Albany, CA)
Data analysis	MSI software (BTI), Curry 7 and 8 Neuroimaging Suite (Compumedics Neuroscan, Compumedics USA, Ltd., Charlotte, NC, USA), Matlab (MathWorks Inc., Natick, MA, USA), SPSS (SPSS Inc., Chicago, IL, USA), ERPSS (Event-Related Potential Laboratory, University of California San Diego, La Jolla, CA, USA), FreeSurfer (version 5.1.), FSL (http://www.fmrib.ox.ac.uk/fsl/)

For manuscripts utilizing custom algorithms or software that are central to the research but not yet described in published literature, software must be made available to editors and reviewers. We strongly encourage code deposition in a community repository (e.g. GitHub). See the Nature Research guidelines for submitting code & software for further information.

Data

Policy information about availability of data

All manuscripts must include a data availability statement. This statement should provide the following information, where applicable:

- Accession codes, unique identifiers, or web links for publicly available datasets
- A list of figures that have associated raw data
- A description of any restrictions on data availability

The datasets generated during and/or analyzed during this study are available from the corresponding author on reasonable request. Source data underlying the graphs and charts presented in the main figures are made freely accessible at https://osf.io/chbnd/.

Field-specific reporting

Please select the one below that is the best fit for your research. If you are not sure, read the appropriate sections before making your selection.

☒ Life sciences ☐ Behavioural & social sciences ☐ Ecological, evolutionary & environmental sciences

For a reference copy of the document with all sections, see nature.com/documents/nr-reporting-summary-flat.pdf

Life sciences study design

All studies must disclose on these points even when the disclosure is negative.

Sample size	The number of participants as well as the number of trials per experimental condition (> 200) was chosen according to Luck, S.J., 2005. An Introduction to the Event-Related Potential Technique. The MIT Press, Cambridge, MA.), and previous work investigating GFBA components in the EEG/MEG (Bartsch et al. 2015, Cereb. Cortex 25, 2828–41; Bondarenko et al., J. Neurosci. 32, 15284–95). Based on EEG data of a similar experiment (Bartsch et al. 2015, experiment 3), we expected the GFBA effects of interest to be at least of medium effect size, for which G*Power (Faul et al., 2007, Behavior Research Methods 39, 175–191) calculations would suggest twenty subjects to be sufficient for our within-subject repeated measures design (Cohen's f > 0.25, power level 0.8, significance level 0.05).
Data exclusions	No data were excluded.
Replication	GFBA modulations for color as assessed by the unattended probe paradigm here have been replicated in previous experiments including a comparable number of participants (cf. Bartsch et al. 2015, Bartsch et al., 2017, Bartsch et al. 2018).
Randomization	There were no different experimental groups.
Blinding	Since participants were not assigned to specific groups, blinding was not necessary.

Reporting for specific materials, systems and methods

We require information from authors about some types of materials, experimental systems and methods used in many studies. Here, indicate whether each material, system or method listed is relevant to your study. If you are not sure if a list item applies to your research, read the appropriate section before selecting a response.

Materials & experimental systems

n/a	Involved in the study
☒	☐ Antibodies
☒	☐ Eukaryotic cell lines
☒	☐ Palaeontology and archaeology
☒	☐ Animals and other organisms
☐	☒ Human research participants
☒	☐ Clinical data
☒	☐ Dual use research of concern

Methods

n/a	Involved in the study
☒	☐ ChIP-seq
☒	☐ Flow cytometry
☒	☐ MRI-based neuroimaging

Human research participants

Policy information about studies involving human research participants

Population characteristics	Twenty-two volunteers participated (mean age 26.0 years, age range 22-33 years, 12 female, all right-handed). All participants had normal or corrected-to-normal visual acuity and reported normal color vision.
Recruitment	Participants were recruited using a lab-intern data base containing mainly students of the Otto-von-Guericke University of Magdeburg.
Ethics oversight	The experimental methods and procedures were approved by the ethics board of the Otto-von-Guericke University of Magdeburg.

Note that full information on the approval of the study protocol must also be provided in the manuscript.
